# Evaluation of a decision aid for women with breech presentation at term: a randomised controlled trial [ISRCTN14570598]

**DOI:** 10.1111/j.1471-0528.2006.01206.x

**Published:** 2007-01-08

**Authors:** N Nassar, CL Roberts, CH Raynes-Greenow, A Barratt, B Peat

**Affiliations:** a Centre for Perinatal Health Services Research, University of SydneyNew South Wales, Australia; b School of Public Health, University of SydneyNew South Wales, Australia; c Department of Perinatal Medicine, Women's and Children's HospitalNorth Adelaide, Australia

**Keywords:** Breech presentation, decision aid, patient information, pregnancy, randomised controlled trial

## Abstract

**Objectives:**

To evaluate the effectiveness of a decision aid for women with a breech presentation compared with usual care.

**Design:**

Randomised controlled trial.

**Setting:**

Tertiary obstetric hospitals offering external cephalic version (ECV).

**Population:**

Women with a singleton pregnancy were diagnosed antenatally with a breech presentation at term, and were clinically eligible for ECV.

**Methods:**

Women were randomised to either receive a decision aid about the management options for breech presentation in addition to usual care or to receive usual care only with standard counselling from their usual pregnancy care provider. The decision aid comprised a 24-page booklet supplemented by a 30-minute audio-CD and worksheet that was designed for women to take home and review with a partner.

**Main outcome measures:**

Decisional conflict (uncertainty), knowledge, anxiety and satisfaction with decision making, and were assessed using self-administered questionnaires.

**Results:**

Compared with usual care, women reviewing the decision aid experienced significantly lower decisional conflict (mean difference −8.92; 95% CI −13.18, −4.66) and increased knowledge (mean difference 8.40; 95% CI 3.10, 13.71), were more likely to feel that they had enough information to make a decision (RR 1.30; 95% CI 1.14, 1.47), had no increase in anxiety and reported greater satisfaction with decision making and overall experience of pregnancy and childbirth. In contrast, 19% of women in the usual care group reported they would have made a different decision about their care.

**Conclusions:**

A decision aid is an effective and acceptable tool for pregnant women that provides an important adjunct to standard counselling for the management of breech presentation.

*Please cite this paper as:* Nassar N, Roberts C, Raynes-Greenow C, Barratt A, Peat B, on behalf of the Decision Aid for Breech Presentation Trial Collaborators. Evaluation of a decision aid for women with breech presentation at term: a randomised controlled trial [ISRCTN14570598]. BJOG 2007;114:325–333.

## Introduction

There is good evidence available from systematic reviews that highlight effective management options for women with breech presentation at term (≥37 weeks). Planned caesarean section has been shown to be the safest form of delivery for women with persisting breech presentation;^1^ however, studies show that planned caesarean section is not without risk for mother and baby in current and future pregnancies,^2^–^4^ and that over 90% of women prefer a vaginal delivery.^5^–^7^ A safe and effective way for women with a breech presentation to reduce the likelihood of noncephalic birth and caesarean section is with external cephalic version (ECV), with recent studies highlighting no increased risk of complications after ECV.^8^–^10^ Each of these options has benefits and risks, and the importance of these varies for each woman, subject to her own personal values and preferences, a situation where a decision aid may be helpful.^11^

Decision aids are practical tools that provide heathcare information for women and their carers to make informed decisions based on unbiased and high-quality research evidence.^11^ Decision aids are nondirective in that they do not aim to steer the user towards any one option, but rather aim to support decision making, which is informed and consistent with personal values. They are also not intended to increase or decrease intervention rates but act as an adjunct to care. A systematic review comparing decision aids with usual care has shown that they improve patient knowledge, create more realistic expectations about outcomes, reduce decisional conflict (uncertainty about a course of action) and stimulate women to be more active in decision making, without increasing anxiety.^11^

Given the evidence, the decision aid was developed based on the option that women must decide whether or not to try ECV to increase the likelihood of a vaginal birth, or otherwise, plan a caesarean section for persisting breech presentation. It was designed to provide information about the benefits and risks of ECV and outcomes of persisting breech presentation that would help prepare women for an informed discussion with their pregnancy care provider. The aim of this study was to evaluate the effectiveness of the decision aid to facilitate informed decision making in a randomised controlled trial among women with a breech presentation at term.

## Methods

The trial was conducted at four Australian tertiary obstetric hospitals that offered ECV, with the study protocol approved by the institutional ethics committee at each hospital. Women with a singleton pregnancy diagnosed antenatally with a breech presentation from 34 weeks of gestation, clinically eligible for ECV and able to read and write English were eligible for the study. The exclusion criteria included contraindications to ECV such as women presenting with a breech in labour, multiple pregnancy, previous caesarean section, severe fetal anomaly, ruptured membranes and indications for caesarean section anyway. All the women provided written informed consent before entry to the study.

Women recruited to the study were randomised to either receive the decision aid in addition to their usual care or usual care only. Usual care involved standard counselling and information on the management of breech presentation from the usual antenatal care provider, an obstetrician or registrar. As this was a pragmatic trial, we made no attempt to standardise usual care, and this was dependent on the individual clinician providing counselling. To avoid the potential for clinicians to impart conflicting information to that in the decision aid, we provided them with an information sheet, at the beginning of the study, detailing a summary of the evidence included in the decision aid.

### Intervention

The decision aid, *Making choices: options for a pregnant woman with a breech baby* (© University of Sydney, 2003), was based on the Ottawa Health Decision Framework^12^ for decision aids and comprised a 24-page booklet, 30-minute audio-CD and worksheet. Evidence about the safety, effectiveness and outcomes of ECV and persisting breech presentation was synthesised from a systematic review of the evidence to provide unbiased, high-quality information about management options.^9^ Information about the content of the decision aid is detailed elsewhere, but briefly it was designed to incorporate information on breech presentation and ECV, probabilities of outcomes tailored to personal risk factors, an explicit values clarification exercise, examples of other patients’ decision-making process and guidance in the steps of decision making.^13^ The decision aid booklet and accompanying audio file can be accessed at the following websites: http://www.health.usyd.edu.au/shdg/ or www.psanzpnmsig.org/impact/. The decision aid was designed in a format that could be taken home and discussed with a partner, and it was produced in English for use by women with a reading age of at least 12 years.^13^ Development of the decision aid involved an iterative process of review and revision with a multidisciplinary project group and content review by international experts. We also conducted two rounds of pilot testing to develop a version suitable for evaluation, including assessment of the acceptability of the decision aid materials.^13^

### Procedure

Eligible women were identified during routine antenatal care and referred to a research midwife for recruitment and randomisation. All the participants completed a baseline questionnaire, and were asked to return to the clinic in 1 week for standard counselling. Women randomised to the study group received the decision aid to take home to review. At the next antenatal visit, women in the study group reviewed the decision aid and worksheet with the research midwife. All women received standard counselling from their clinician and then completed a first follow-up questionnaire following their consultation. At 3 months postpartum, a second follow-up questionnaire was mailed with reply paid envelopes to all the participants. Information on pregnancy and birth outcomes was obtained from the obstetric records of all women.

Treatment allocation was randomly generated using computer and stratified by parity and centre using random variable block sizes. Participants were randomised by telephoning a remote, central location. It was not possible to blind women to allocation group; however, to minimise contamination, a research midwife was employed at each centre, and antenatal staff were kept blinded to the treatment allocation and the actual content of the decision aid.

### Outcomes

The effectiveness of the decision aid to improve patient decision making was determined by assessing women's knowledge and decisional conflict. Knowledge of management options and outcomes for breech presentation was assessed by asking women true/false questions at baseline and at first follow up. The measure included 20 questions related to general knowledge about breech, ECV and benefits and risks of ECV and persisting breech presentation. Decisional conflict refers to uncertainty about a course of action, and in our case, it was related to the decision of whether or not a woman chose to try ECV.^14^ The Decisional Conflict scale (low literacy version), a 10-item scale was used to measure uncertainty and specific factors such as feeling uninformed, unclear about values and unsupported in decision making.^14^ Each item contained a 3-point Likert scale that was scored between 1.5 (low decisional conflict) and 4.5 (high decisional conflict) and then items were summed and standardised to a score between 1, representing low decisional conflict, and 100, extreme decisional conflict. This measure was assessed at each stage of data collection.

A number of affective (anxiety, satisfaction and participation in decision making) and behavioural outcomes (intention and actual decision taken and acted upon) were also examined.^15^ The six-item short form of the State scale of the Spielberger State–Trait Anxiety Inventory was used to measure anxiety,^16^ Satisfaction With Decision scale was applied to assess patient satisfaction with healthcare decisions,^17^ women's attitudes of the importance of undergoing an ECV were assessed using the attitude scale based on components adopted from the Theory of Planned Behaviour,^18^ values and choice predisposition measures were adapted from pre-existing scales developed by the Ottawa Health Decision Centre (© O’Connor A; Cranney 2000; www.ohri.ca/decisionaid) and women's preferred role in decision making was ascertained using the five-item Degner Control Preferences scale.^19^ All the measures adapted from pre-existing scales were revalidated during pilot testing of the decision aid.^13^

Although the decision aid was not intended to influence rates of intervention, we also collected secondary outcomes to assess the use of health service such as ECV uptake and maternal and perinatal outcomes. Brief socio-demographic data were collected to assess the comparability of the two groups. Compliance and acceptability of the decision aid materials was also assessed for women randomised to the study group. Compliance in the form of optimal use was considered when women used all three components of the decision aid, had reviewed the audio-guided workbook and audio-CD and completed the worksheet. General comments regarding the application, acceptability and recommendation of the decision aid materials to other women was also ascertained.

### Sample size and statistical analyses

Sample size was based on results from a systematic review comparing decision aids with usual care interventions.^11^ To detect a similar significant mean difference in decisional conflict scores (−5.75 out of 100; 95% CI −8.63 to −2.87; median standard deviation 13.25) and knowledge scores (18.75 points out of 100; 95% CI 13.1–24.4; median standard deviation 20) of women, a sample size of 84 women in each arm of the trial was required (significance 0.05, power 0.8).^11^ To allow for loss to follow up, the sample size was inflated to give an effective sample size of 100 women per arm of the trial and 200 women in total.

All data analyses of group differences were conducted according to intention to treat and were calculated at each appropriate data collection point. Results for knowledge outcomes were analysed by summing and calculating the percentage of correct responses for each individual. Scoring for affective outcome measures was calculated according to recommended algorithms,^14^,^17^–^20^ and the ranges are presented for each outcome measure in the results tables. Group differences in categorical outcomes were assessed using chi-square or Fisher's exact tests, with relative risks and associated 95% CI calculated.^21^ Continuous variables were examined using two-sample *t* tests with Satterthwaite correction applied in cases with unequal variances.^22^ Repeated measures analysis of variance was conducted to assess group differences in outcomes over time. Two-sided *P* values less than 0.05 were regarded as statistically significant, and all data were analysed using SAS version 8.2 (SAS Institute, Cary, NC, USA).

## Results

**Figure 1:**
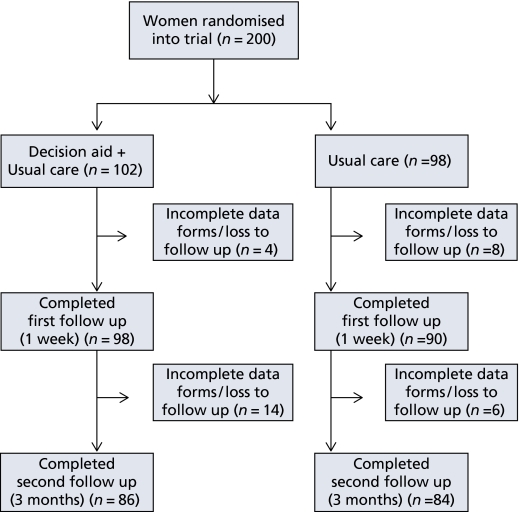
Flow of study participants throughout trial.

A total of 200 women were recruited and randomised to the trial between June 2003 and January 2005 (Figure 1). Twelve women were lost to follow up due to onset of labour before first follow up or incomplete data forms, giving an effective sample of 188 (94%) women. There were no differences in maternal age, level of education, parity or treatment allocation between responders and nonresponders. At 3 months postpartum, the response rate for second follow-up assessment was 85%, with no difference in the rate of loss to follow up between the two groups (*P* = 0.77).

Maternal characteristics and baseline measures of cognitive and affective outcomes were comparable between groups (Table 1). The majority of women reported a preference for vaginal birth (>90%) as they believed this to be the most natural mode of delivery. Eighty percent of respondents had heard of a procedure that may facilitate the turning of a breech presentation to a head-down position, and two-thirds stated that they would consider trying it (Table 1).

**Table 1:** Baseline maternal characteristics

Maternal characteristics	Decision aid (*n* = 102)	Usual care (*n* = 98)
Maternal age in years, mean (range)	31.3 (16–44)	30.7 (20–41)
Gestational age at recruitment in weeks, mean (range)	36.0 (34–39)	36.1 (34–38)
Nulliparous (%)	63.4	70.1
Education (%)		
Secondary	29.0	25.8
Post-secondary	71.0	74.2
Preference for vaginal delivery (%)	91.0	94.8
Heard of external cephalic version (%)	80.4	81.3
Knowledge of caesarean section as safest mode of delivery for breech presentation (%)	72.0	71.1

Table 2 presents primary outcomes for women in the decision aid group compared with women in the usual care group after intervention. Women who reviewed the decision aid had significantly higher knowledge scores and significantly lower decisional conflict scores compared with women in the usual care group ([mean difference 8.4; 95% CI 3.1, 13.7], *P* < 0.01 and [mean difference score −8.9; 95% CI −13.2, −4.7], *P* < 0.01, respectively). In this case, women in the decision aid group felt significantly more informed and experienced greater certainty about their decisions. They also had clearer values; felt more supported and felt that they had made more effective choices (Table 2). The change in knowledge (*P* < 0.001) and decisional conflict scores (*P* = 0.01) before and after intervention were also significantly greater for women who reviewed the decision aid than for women who received usual care only. The trend in results was consistent when stratified by maternal age, parity and education, however, due to small numbers they were not statistically significant (not presented).

**Table 2:** Cognitive, affective and behavioural outcomes

Cognitive, affective and behavioural outcomes	Decision aid (*n* = 102)mean (SD)	Usual care (*n* = 98)mean (SD)	Mean difference (95% CI)
Decisional conflict (1–100, 1 = low decisional conflict			
Baseline	45 (29.0)	43 (27.5)	2.74 (−6.56, 12.05)
First follow up	4.6 (9.0)	13.5 (19.2)	−8.92 (−13.18, −4.66)
Second follow up	4.2 (12.5)	12.7 (20.9)	−8.49 (−13.69, −3.29)
**Knowledge (% correct responses)**			
Baseline	69 (28.7)	69 (25.8)	−0.46 (−8.25, 7.33)
First follow up	88 (19)	79 (18)	8.40 (3.10, 13.71)
**Satisfaction with decision making (6–30, 6 = low satisfaction)**			
First follow up	26.3 (3.9)	25.6 (4.1)	0.64 (−0.53, 1.81)
Second follow up	27.7 (3.0)	26.2 (3.6)	1.45 (0.44, 2.46)
**Anxiety (20–80, 20 = low anxiety), *n*** (%)			
Baseline	45.8 (15.0)	47.4 (13.9)	−1.65 (−5.73, 2.42)
First follow up	41.4 (12.5)	44.4 (13.9)	−2.97 (−6.78, 0.84)
Second follow up	29.2 (9.9)	30.8 (10.5)	−1.66 (−4.76, 1.44)
Positive attitude towards ECV (%)*	62.5	44.3	RR 1.41 (1.07, 1.85)
**Values (1–7, 7 = very important)**			
Turning breech baby by ECV	5.3 (1.9)	4.5 (2.1)	0.74 (0.17, 1.32)
Adverse effects of ECV	4.4 (1.8)	3.8 (2.0)	0.58 (0.03, 1.13)
**Enough information to make decision (%)**			
First follow up	95.7	73.6	RR 1.30 (1.14, 1.47)
Second follow up	90.0	77.0	RR 1.16 (1.02, 1.33)
**Intention for ECV (%)**			
Baseline	74.0	66.0	RR 1.12 (0.93, 1.35)
First follow up	77.1	55.7	RR 1.38 (1.12, 1.70)

* Proportion above overall median (18.5) = positive attitude.

At first follow up, the proportion of women who considered having an ECV was significantly different between the two groups (77 versus 56%, *P* = 0.002) (Table 2). After standard counselling alone, women who would consider having an ECV declined from 66 to 56% (*P* = 0.15). A further 14% of women remained undecided about their decision. For women receiving the decision aid, most considering ECV maintained their intention (from 74% of women before to 77% after reviewing the decision aid), and those initially unsure chose not to have an ECV. Only 1% of women were still unsure about their decision after reviewing the decision aid. Women in the decision aid group were significantly more likely to report that they had enough information to make a decision regarding the management of their breech presentation compared with women in the usual care group at each point of follow up (*P* < 0.01) (Table 2).

At 3 months postpartum, the majority of women in the decision aid group (90%) reported that they had enough information to make their decisions, and only 8% would have made a different decision. In contrast, only 77% of women who received standard counselling alone felt informed and certain about their decisions regarding the management of their breech baby (Table 2). In addition, 19% reported that, upon reflection, they would have made a different decision. Women who reviewed the decision aid were more likely to feel satisfied with their decisions, and they experienced significantly greater satisfaction with their overall experience of pregnancy and childbirth compared with women in the usual care group (76 versus 65%, *P* = 0.03).

No differences were found in the level of anxiety among women in the two groups at baseline, first or second follow up; although, there was a large decline in anxiety among women in both groups at 3 months postpartum (Table 2). Most women preferred to take an active role in decision making; 28% wanted to make their own decisions and 71% wanted to make their decisions collaboratively with their pregnancy care provider, with results similar for the two groups and at each point of data collection.

Of the women in the decision aid group, a 93% compliance rate was reported in the optimal review of the decision aid materials. Three-quarters of women reviewed the decision aid with a partner or family member, and 87% discussed the decision aid and their decision with a significant other. Median time women spent reviewing the decision aid with a research midwife was 10 minutes. Women reported the decision aid to be helpful, clear and easy to understand, and 99% would recommend it to other women facing a similar decision.

Despite the disparity between the two groups in their intention for ECV (Table 2) there was no significant difference in the proportion of women who underwent the procedure. Among women who did not have an ECV, women in the usual care group were more likely to decline ECV (65 versus 46%, *P* = 0.11), most commonly because of concerns about the safety of the procedure. In contrast, women in the decision aid group were more likely to want ECV (77 versus 56%), but 30% did not have one (Table 3). Reasons for women in the decision aid group not having ECV are given in Table 3. Of the women who did undergo ECV, the overall success rate was similar for the two groups (mean = 48%). There were no differences in presentation at birth, mode of delivery, maternal and perinatal outcomes for women in the decision aid and usual care group (Table 3).

**Table 3:** Pregnancy and birth outcomes

Pregnancy and birth outcomes	Decision aid (*n* = 98)	Usual care group (*n* = 90)	*P* value
**ECV, *n* (%)**			
Yes	52 (53)	51 (57)	0.72
No	42 (43)	37 (41)	
**Reason for no ECV, *n* (%)**	*n* = 42	*n* = 35	
Refused	19 (45)	24 (69)	0.11
Advised against ECV			
Bleeding, rupture of membranes	7 (17)	2 (6)	
Clinician advice	7 (17)	3 (9)	
Spontaneous version	9 (21)	6 (16)	
**Reason for refusal of ECV (%)**			
It took up too much time	0	8	0.24
Not safe enough for baby	61	88	0.03
Not safe enough for mother	56	65	0.51
Results not high enough to try	76	84	0.50
Vaginal delivery not guaranteed	42	50	0.57
Prefer caesarean section	44	16	0.03
**ECV success (*n* = 103), *n* (%)**	22/52 (42)	27/51 (53)	0.28
**Presentation at birth, *n* (%)**			
Cephalic	33 (34)	32 (36)	0.74
Noncephalic	64 (66)	56 (64)	
**Mode of delivery, *n* (%)**			
Vaginal (cephalic presentation)	33 (34)	29 (34)	0.92
Planned caesarean section, no labour	47 (48)	41 (48)	
Planned caesarean section, with labour	11 (11)	13 (15)	
Caesarean section during labour	6 (6)	5 (6)	
**Apgar scores, *n* (%)**			
Apgar1 > 7	91 (91)	83 (92)	0.87
Apgar5 > 7	96 (98)	88 (98)	0.93
**Sex, *n* (%)**			
Male	41 (42)	39 (44)	0.84
Female	57 (58)	51 (56)	
**Gestational age >37 weeks**	93 (96)	85 (98)	0.49
**Birthweight in g, mean (SD)**	3364 (493)	3325 (419)	0.58
**Maternal length of stay in days, mean (SD)**	4.7 (3.6)	4.7 (1.7)	0.89

Numbers may not add up to totals due to missing data.

## Discussion

This is the first study to assess the effectiveness of a decision aid for women with a breech presentation at term. Findings show that women found the decision aid to be an effective, useful and acceptable adjunct to standard counselling about the management options for breech presentation. Compared with usual care, women who reviewed the decision aid felt significantly more informed, experienced less uncertainty and made decisions that were consistent with their personal values and preferences, without increased anxiety. Subsequently, these women experienced increased satisfaction with their decisions and expressed greater satisfaction in their overall experience of pregnancy and childbirth. The decision aid was also well received with an overwhelming majority of participants recommending it to other women facing a similar decision.

The strengths of the study include the randomised trial design with sufficient power to show changes in primary outcomes of decisional conflict and knowledge. Recruitment of women at the point of decision making, inclusion of a usual care arm and assessment of baseline predispositions and follow up of participants 3 months postpartum all increased the generalisability of the study findings.^23^ Blinding of clinicians and employment of a research midwife to interact with women also minimised contamination of women and care providers. Information included in the aid was based on seven systematic reviews of the management options for breech presentation at term^1^,^8^,^24^–^28^ and incorporation of effective evidence-based strategies for presenting patient information and risk communication.^20^,^29^–^31^

Findings show that the decision aid is effective in increasing informed decision making, with few women remaining undecided about their decision after use. In contrast, usual care alone, while it had some positive effects in improving women's knowledge about breech presentation and reducing levels of decisional conflict, led to a large proportion of women remaining undecided about their decisions or choosing not to have an ECV after counselling. Of particular concern was the relatively large proportion of women (30%) who intended to have ECV and who then did not have one. These findings suggest that some clinicians may not be fully informed of current evidence for breech presentation, or more likely may not support ECV at all. A recent survey of obstetricians regarding the management of breech pregnancies in Australia and New Zealand found almost one-third do not recommend ECV to their patients.^32^ Of those obstetricians practicing ECV, a number of them were found to be undertaking practices for which safety has yet to be demonstrated, including 28% carrying out ECV outside hospitals and 42% conducting ECV before 37 weeks of gestation.^32^ Furthermore, the constant rate of term breech births in Australia suggest there has been limited uptake of ECV.^33^ To ensure optimal application of the decision aid, clinicians must have a strong commitment to the reduction of breech presentation and support ECV as a safe and effective procedure in reducing noncephalic births.^34^ Barriers to the practice of ECV, particularly improving knowledge and training of clinicians need to be addressed to ensure that women are provided with current, evidence-based counselling and options for the management of breech presentation at term.

The implementation of the decision aid in obstetric settings could provide a tool for overcoming some of the barriers to ECV experienced by clinicians. It could increase evidence-based practice through the influence of patient choice on clinicians and by also providing, not only to women but also to care providers, a standard source of information that may facilitate consistent counselling and practice. As clinicians were blinded as to whether their patients received the decision aid or not, acceptability by clinicians was not assessed in the current study. Further research to assess the usefulness, acceptability and implementation of decision aids by pregnancy care providers is essential to ensure optimal application. Assessment of clinicians’ perceptions of decision aids for other healthcare issues has shown that practitioners found the decision aid intervention acceptable and helped in a majority of consultations.^35^,^36^ Training for clinicians in principles and practice of decision aids and informed decision making has also been shown to help overcome implementation issues.^37^

Overall, the positive responses and high level of compliance among participants found both in this trial and in the pilot study of the decision aid^13^ suggests that the decision aid may be a feasible adjunct to usual care in obstetric settings. The time available during consultations is often constrained, and the decision aid may be an efficient tool in preparing women for an informed and focused discussion with their care provider. This is particularly important as 95% of women expressed a preference for involvement in decision making, which is an important factor in their overall satisfaction with care.^38^,^39^ In addition, patients may have a limited attention span, find new information difficult to recall and may understand as little as half.^40^ Thus, the audio-guided workbook and take-home format of the decision aid provides women the opportunity to review, consider and discuss their options with their partner and/or family in a self-paced, active way at their own convenience at home.

This is the first study to develop and evaluate a decision aid for women with a breech presentation in late pregnancy. One previous study developed a pamphlet for women with a breech pregnancy as a part of a series of eight maternity evidence-based leaflets. However, as this information was dated and the leaflets evaluated collectively, it was difficult to draw any comparisons.^41^ Nevertheless, findings from this study are consistent with results from trials evaluating the effectiveness of decision aids for other pregnancy care issues such as prenatal testing and vaginal birth after caesarean section.^42^–^44^

Some potential limitations of the study are that the applicability of the decision aid is only relevant in obstetric hospitals that offer ECV. Generalisability of the findings may also be limited to women fluent in English. However, with the positive findings there is the potential for the decision aid to be translated into other languages. The audio-CD also improves access to women with poor literacy and those from non-English speaking backgrounds as it has been shown that their spoken vocabulary is often at a higher level than their written vocabulary.^45^ A further limitation is the continuing commitment and costs associated with maintaining current and up-to-date information in the decision aid. However, availability on the Internet could overcome these weaknesses and make it more accessible to women, easier to update and less expensive to maintain. In its current format, the audio component adds considerable length and complexity to the development and cost of the decision aid. Thus, we are currently evaluating a decision aid for pain relief in labour that compares a workbook with and without an audio-CD to assess the relative benefit of the audio component.^46^

## Conclusion

In conclusion, a decision aid for women with a breech presentation at term is an effective, useful and acceptable tool that provides an important adjunct to usual care. It also supports informed decision making, which is a strong predictor of satisfaction with care in pregnancy and childbirth. To ensure optimal implementation of the decision aid, barriers to the promotion and practice of ECV by care providers need to be identified and addressed. The results of this study show that the application of decision aids has the potential to improve consumer information and participation in clinical decisions across a wide spectrum of pregnancy care.
